# Female Antibodies Go the Distance against Influenza Viruses

**DOI:** 10.1128/mbio.02106-22

**Published:** 2022-09-12

**Authors:** Marina R. Good, Jenna J. Guthmiller

**Affiliations:** a Department of Immunology and Microbiology, University of Colorado Anschutz Medical Campus, Aurora, Colorado, USA; b Immunology Graduate Program, University of Colorado Anschutz Medical Campus, Aurora, Colorado, USA

**Keywords:** biological response modifiers, humoral immunity, influenza, influenza vaccines, neutralizing antibodies

## Abstract

Females have long been described to generate superior humoral immune responses relative to those in males. In the article by Ursin et al. (R. L. Ursin, S. Dhakal, H. Liu, S. Jayaraman, et al., mBio 13:e01839-22, 2022, https://doi.org/10.1128/mbio.01839-22), the authors showed that female mice generated a more robust, broadly reactive, and protective humoral immune response against influenza viruses in comparison to their male counterparts. Female mice demonstrated more efficient germinal center responses, including increased class switching and affinity maturation. Therefore, sex plays an important role in acquisition of protection against influenza viruses by modulating the generation of protective B cell responses. In this commentary, we dive into how this study builds on our understanding of how females generate superior antibody responses against influenza viruses and how this informs vaccine design.

## COMMENTARY

Influenza viruses remain a major public health problem and are a leading cause of respiratory infections, hospitalization, and deaths in the United States. While seasonal influenza vaccines provide modest protection against influenza viruses, host factors play an important role in how humans respond to infection. Importantly, sex remains one of the most important biological variables in determining how well humans respond to the vaccination and are protected from infection. Previous data on immune responses after vaccination demonstrated that there are sex-related differences, with females generating superior immunity following vaccination (1, 2). However, the mechanisms by which females generate improved responses to vaccination remain ill-defined. Understanding how sex affects immunity is an important goal that will improve vaccine immunogenicity and effectiveness.

In the study by Ursin et al. (3), the authors investigated the breadth of the humoral immune response in female versus male mice by using mutant influenza viruses. The authors found that female mice were better protected against influenza viruses and generated a broader antibody response, both of which are linked to bigger and better germinal center responses. Here, we discuss how these findings fit into and expand upon our current understanding of sex as a determinant of how females generate superior antibody responses, with implications for improving vaccine effectiveness across sexes.

## FEMALES MOUNT A MORE ROBUST ANTIBODY RESPONSE

Sex has long been a determinant of how well healthy adult humans respond to vaccination, with females generating higher humoral and cellular immune responses following vaccination with nearly every vaccine studied (4). This is due to the fact that females are more likely to generate a robust inflammatory response relative to males, which leads to more immune cell activation, antigen presentation, and selection. This increase in inflammatory responses is due in part to sex-related environmental factors, such as estrogen levels, as well as genetic factors, particularly the possession of two X chromosomes.

Estrogen has been well documented in regulating a variety of immune functions, including B and T cell development, cellular activation and trafficking, cytokine and chemokine production, and cellular survival (5), all of which can impact humoral immunity. Moreover, several key B cell-regulating genes are on the X chromosome, including *Tlr7*, which is frequently overexpressed in females due to incomplete inactivation of one X chromosome (6). Notably, Toll-like receptor 7 (TLR7) expression by B cells promotes IgG2c class switching and protective humoral immunity against viruses (7, 8). Ursin et al. identified that females had improved IgG2c class switching relative to that in male mice. Together, the impacts of estrogen and elevated TLR7 expression can lead to increased type 1 inflammatory cytokines, including type I interferons, and B cell activation and class switching to IgG2c.

## FEMALES GENERATE BETTER GERMINAL CENTER RESPONSES

In the article, the authors found that females generated a greater germinal center response, leading to increased efficiency of somatic hypermutation (3). These data suggested that not only do females induce a greater germinal center-derived antibody response, but also that this response is qualitatively better. The observed phenotype could be due to both genetic and hormonal factors. First, a recent report showed that male B cells are poorly positioned within the B cell follicle in a sex-dependent manner, leading to impaired humoral immunity against both foreign and self antigens (9). As a result, female B cells are better positioned to enter germinal centers and to be presented antigen. In addition, TLR7 stimulation can lead to more and better germinal center B cells, which can increase somatic hypermutations and affinity maturation (8). Although these mechanisms were not explored in the study by Ursin et al. (3), these data suggest that sex differences could lead to differences in germinal center responses and B cell selection via sex-dependent environmental and genetic factors that regulate B cell positioning and activation.

## FEMALES PRODUCE A BROADER ANTIBODY RESPONSE

Perhaps the most important finding of the Ursin et al. study (3) was that influenza virus-specific antibodies targeted more epitopes and were more cross-reactive across influenza virus subtypes. This is an important finding, as the factors that regulate the quantity and quality of influenza-specific antibodies (e.g., hormones, TLR7) may also regulate the breadth of the antibody response. As humoral immunity against influenza viruses typically targets the parts of the virus that tend to mutate, these data suggest that females are inclined to generate responses against portions of the virus that are more conserved and that provide better protection against distantly related viruses. Moreover, the antibody response of the female mice was directed toward a variety of epitopes, possibly limiting intrahost viral evolution, as pressure at multiple sites will mitigate viral evasion of host antibody responses.

In another recent study, broadly neutralizing antibodies against influenza viruses were noted for their capacity to be polyreactive, a phenomenon in which an antibody can bind to multiple molecularly distinct antigens, including self antigens (10). Females are dramatically more likely to have autoimmune disorders, which are frequently characterized and exacerbated by the presence of autoantibodies against self antigens. Moreover, autoimmune diseases, such as systemic lupus erythematous, are characterized by epitope spreading, where the autoantibody response targets more self antigens over time. B cell tolerance mechanisms suppress polyreactive and autoreactive B cells from developing and reacting with self antigens. However, immune tolerance limits the robust induction of broadly protective antibodies against influenza viruses, as recently shown by Sangesland et al. (11).

Mechanistically, estrogen can limit B cell tolerance, thus leading to more autoreactive B cells in the naive B cell repertoire (12). Despite the implications for autoantibody and autoimmune development, defects in suppression of polyreactive and autoreactive B cells may allow for the recruitment of more B cell specificities against numerous epitopes, including highly conserved epitopes of hemagglutinin. Alterations in B cell development and function in females may then lead to superior binding breadth and viral neutralization by the ensuing antibody response. However, this raises the concern that influenza vaccination in females may generate high-affinity self-reactive antibodies, though there is no evidence that influenza vaccination induces pathogenic antibodies. As a result, females may be more primed to generate broadly reactive antibodies against influenza viruses and other foreign and self antigens.

## EQUALIZING IMMUNOGENICITY OF INFLUENZA VACCINES ACROSS SEXES

The work presented by Ursin et al. and other studies describing sex as a biological variable of vaccine response have repeatedly demonstrated that females generate superior antibody responses relative to males. This raises the following question: how do we improve vaccine immunogenicity in males? Certain factors cannot reasonably be changed without significant side effects, such as hormonal levels, but other factors can be manipulated, such as TLR7 stimulation. Consistently, TLR7 has been shown to be an important factor in promoting germinal center responses, affinity maturation, and IgG2c class switching.

A preclinical study using TLR7 agonists as adjuvants for influenza vaccination showed improved IgG2c (or IgG2a for BALB/c mice) class switching relative to that in nonadjuvanted groups (13). Moreover, the same study found that the TLR7-adjuvanted vaccine robustly induced cross-group protection against H3N2, suggesting TLR7 stimulation can induce broadly protective antibody responses. Additionally, a second study showed a TLR7 adjuvant robustly induced a broadly reactive IgG2c antibody response against a wide array of H5 viruses (14). However, both studies only used female mice and had no accompanying evaluation of whether stimulating TLR7 improved immunity across both sexes. Therefore, it is still unknown if targeting TLR7 can increase humoral immune responses in males.

## CONCLUDING REMARKS

In the article by Ursin et al., the authors identified that quantitative and qualitative differences in germinal center responses were responsible for sex-based differences in protective humoral immunity against influenza viruses (3). This study further expands our current knowledge of sex-based differences in humoral immunity by identifying that females generate a broader antibody response, a feature that is important for immunity against antigenically variable pathogens like influenza viruses. By harnessing the factors that improve immunity in females, it will be possible to engineer vaccines that induce broadly protective antibody responses against influenza viruses independent of sex. It is critical to continue to study immune responses in both males and females to ensure that vaccines are providing equitably superior and broader humoral immunity in all recipients.
